# Self-service kiosk for testing sunglasses

**DOI:** 10.1186/1475-925X-13-45

**Published:** 2014-04-25

**Authors:** Marcio M Mello, Victor AC Lincoln, Liliane Ventura

**Affiliations:** 1 Department of Electrical Engineering, Escola de Engenharia de Sao Carlos – University of Sao Paulo/Av. Trabalhador Saocarlense 400, 13566-590, Sao Carlos, SP, Brasil

## Abstract

**Background:**

Sunglasses users may only be assured on their ultraviolet protection by purchasing certified products, however they are not able to check if sunglasses are still ultraviolet (UV) protected as they age, unless they resort themselves to a professional who is qualified for using a spectrophotometer and is acknowledged on the standards for providing a report for the user. Current literature establishes safe limits on the exposure of the eyes relatively to the ultraviolet radiation exposure for the UVA and UVB ranges (280 nm – 400 nm). The UV protection is category dependent. Sunglasses are categorized from 0 to 4 and the categories are determined by the lenses transmission’s percentage on the visible range (380 nm – 780 nm).

**Methods:**

In order to overcome inaccessibility of such measurements on sunglasses, a prototype for testing ultraviolet protection on sunglasses, according to Brazilian Standards, has been developed for amateur use. The system consists of assembling UVA and UVB light sources and two UV responsive photodiode sensors, with Erythema action response for measuring UV protection; for categories measurements, combination of white light and LEDs were used for the visible range, as well as a light sensor having spectral response similar to the human eye. Electronics has been developed for controlling the measurements and software has been implemented for providing the report as well as for the user’s interface.

**Results:**

All the system was embedded as a self-service touch screen kiosk and provides transmittance measurements that are within the deviation limit required by NBR15111, i.e., 0.25%. Measurements were performed in over 45 sunglasses and compared to CARY 5000 – VARIAN spectrophotometer and present a good correlation for the measurements of transmittance in the visible spectral range (r^2^ = 0.9999) and in the ultraviolet range (r^2^ = 0.9997).

**Conclusions:**

The prototype identifies the UV protection, for non-corrective sunglasses, according to category of the lens and is available for the public. In addition to educating the population about the importance of wearing protected sunglasses, the prototype has also allowed the public to have access to information about the quality of protection of their own sunglasses in an easy and free testing method.

## Background

It is still controversial in literature the harms of UV radiation for each component of the ocular media. The corneal epithelium absorbs UV-A and UV-B radiation, avoiding inner eye components from extreme radiation, but severe exposure to UV radiation may induce corneal changes [1,2]. Yet, a number of ocular diseases in the internal structures of the eye are related to UV radiation exposure, such as cataract [3].

Sunglasses can attenuate ocular exposure, but unprotected sunglasses and its incorrect use, may interfere on this attenuation [4]. Sunglasses can increase UV exposure of the crystalline lens and corneal limbus by disabling the eyes’ natural mechanisms of lid closure and pupil constriction [5].

Sunglasses are world widely used, whether for glamour, visual comfort and particularly for ocular protection, for those who are aware that excessive ultraviolet radiation may harm the eye if lenses are not properly protected [6,7]. Global standards specify physical properties (mechanical and optical) for sunglasses and sun glare filters of nominal plane power lenses (nonprescription lenses), which are intended for protection against solar radiation for general use, such as social and domestic purposes, including road use and driving. Among many required testing items for safety, the ones that we have implemented in the prototype of this work refers to the spectroscopic measurements regarding the transmission measurements of the UVA, UVB and visible range, which are within the 280 nm – 780 nm range. The American Standard [8] and British and European Standard [9] require UV protection on the 280 nm – 380 nm range. Brazilian Standard [10] and The Australian/New Zealand Standard [11] have an extended UVA protection, thus the wavelength range for safety is from 280 nm – 400 nm. Transmittance measures include ultraviolet radiation (UVR) for an effective protection test of spectacles against eye diseases [6,7], infrared radiation, and traffic signal light radiation, so that minimum thresholds required for traffic signs visibility and visible radiation can be established. These measures ensure the minimum safety requirements to the population, indicating excessively dark lenses, which can limit the ability to identify objects in shadows while driving, extremely colored lenses, which can affect the detection and recognition of colors, and filter protection against harmful UV radiation.

Measurements of the UVA and UVB protection for sunglasses are categories of sunglasses reliant. Categories of sunglasses are labelled based on the percentage of visible light transmitted through the spectacles. Visible light transmittance is calculated proportionally to the response of the human eye for the different wavelengths within this range.

Fashion spectacles are categorized by the amount of visible light – 380 nm 780 nm - allowed to pass through the lens. Category rating, ranging from 0 - 4, is given to determine how light or dark sunglasses lenses are, the higher the number, the darker the lens colour. The labelling requirements for sunglasses are based on the transmittance values, given by Table 1.

**Table 1:** **Transmittance for sun glare filters for general use of NBR15111**(**2013**) [10]

	**Requirements**			
	**Ultraviolet spectral range**		**Visible spectral range**	
**Filter category**				
	**Maximum value of spectral transmittance *τ***_**F**_**(*λ*)**	**Maximum value of solar UVA transmittance *τ***_**SUVA**_**(*λ*)**	**Range of luminous transmittance (380-780 nm) *τ***_**V**_	
	**280-315 nm**	**> 315-400 nm**	**From over %**	**To %**
0	0.1 - *τ*_V_	*τ*_V_	80.0	100
1			43.0	80.0
2			18.0	43.0
3		0.5 - *τ*_V_	8.0	18.0
4			3.0	8.0

The luminous transmittance of the sun glare filter for CIE standard illuminant D 65 [12,13], τ_V_ is expressed by:

(1) $$\tau_{v}=\frac{\sum_{\lambda =380}^{780}\tau_{F}\left(\lambda \right).V\left(\lambda \right).S_{D65\lambda }\left(\lambda \right).\Delta \lambda }{\sum_{\lambda =380}^{780}V\left(\lambda \right).S_{D65_{\lambda }}\left(\lambda \right).\Delta \lambda }$$

τ_F_ (λ) is the spectral transmittance of the sun glare filter; S_D65λ_ (λ) is the spectral distribution of radiation of CIE standard illuminant D65 [12,13]; V (λ) is the spectral luminous efficiency for daylight vision [13].

**Figure 1:**
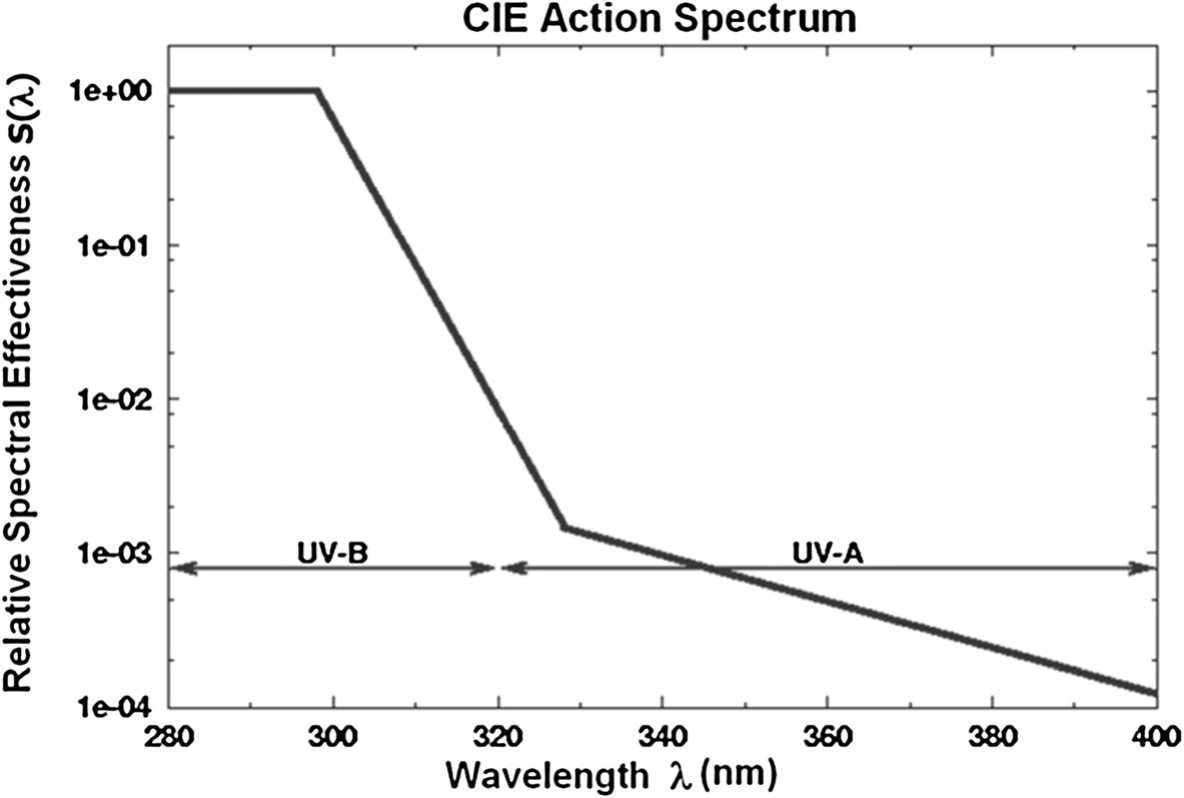
**Graphical representation of the spectral effectiveness of UVR - S (λ) as defined by CIE **[14].

Solar UV-transmittance τ_SUV_ is the mean of the spectral transmittance between 280 nm - 400 nm weighted with the solar radiation E_sλ_ (λ) at sea level for air mass 2 and the relative spectral effectiveness function for UV radiation S (λ) [14] as shown in Figure 1, is given by equation (2):

(2) $$\tau_{\mathrm{SUV}}=\frac{\sum_{\lambda =280}^{400}\tau_{F}\left(\lambda \right).E_{s\lambda }\left(\lambda \right).S\left(\lambda \right).\Delta \lambda }{\sum_{\lambda =280}^{400}E_{s\lambda }\left(\lambda \right).S\left(\lambda \right).\Delta \lambda }$$

## Methods

### Transmittance measurements

The developed self-service kiosk provides measurements of the categories of the sunglasses lenses and their respective UV protection, leading to a final report on whether they are appropriate for use, based on the parameters provided by Table 1. It does not substitute spectroscopic measurements performed on sunglasses for certification purposes, but it provides well-correlated results for the public.

**Figure 2:**
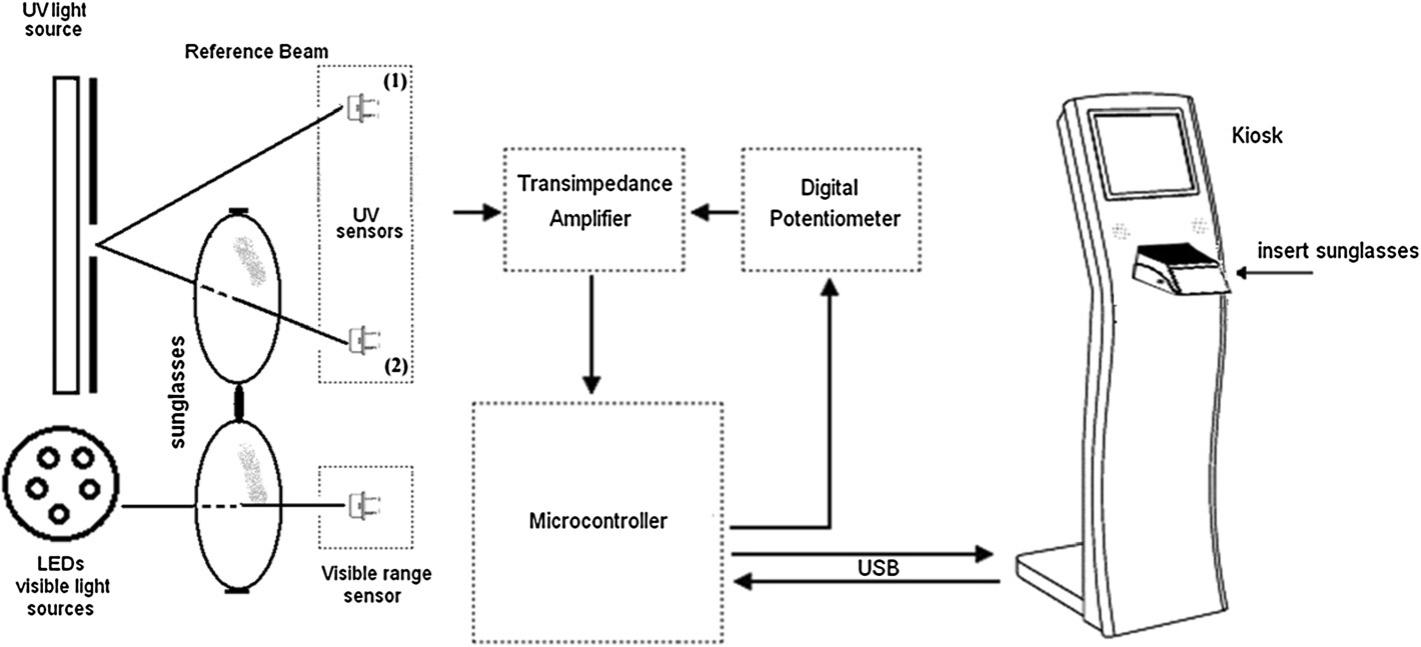
**System**’**s block diagram.**

As an interface software routine, the user places the sunglasses in an appropriate compartment of the kiosk and the category of the sunglasses is measured in one of the lenses as well as the UV protection on the other lens, as shown on the chart in Figure 2. The data is stored and the user is asked to replace the sunglasses in such a way that the category and UV protection measurements are performed for a second time, but now on the opposite lenses than previously done, and finally the report provided to the user is based on both measurements data for each pair of lenses.

The ideal light sources and sensors for each type of measurements would be light sources that would simulate the sun spectrum for the UV range (280 – 400 nm) - E_sλ_ (λ) - as well as for the visible range (380 – 780 nm) - S_D65λ_ (λ). Ideal sensors would be the ones that would respond similarly to the retina for the visible range - V (λ) - and that would have the spectral effectiveness of UVR response - S (λ) - for the ultraviolet range. However, it’s also acceptable to have light sources and sensors as being a combination of the illuminating source and the sensor, in such a way that the resulting product is equivalent to the product E_sλ_ (λ).S (λ) for the UV range; and the product of S_D65λ_ (λ).V (λ) for the visible range. This product should be equivalent for every 5 nm, which is the wavelength interval that spectroscopy should be performed when UV protection on sunglasses are to be tested for certification.

For the category measurements, we have used as illuminating sources a combination of LEDs, with band peaks at the 405 nm; 450 nm; 550 nm; 565 nm; 610 nm and 680 nm and sensors BPW21R. For the UV measurements we have used TL 4W/05 – F4 T5 lamp as the illuminating source and two sensors EPD-365-0-1.4, one being the reference sensor and the other the measuring sensor. The UV sensors’ signals are compared to each other at a rate of 500 measurements per second, so avoiding lamp fluctuation interferences in the results. The dual beam set up improves the control of fluctuations in the system, avoiding undesired errors in the measurements, as learned from previous single beam systems developed from some of the authors of this work [15,16].

### Softwares

Software for the microcontroller board was developed see - Additional file 1 - as well as for the communication of the PC, running Windows’ system, placed inside the kiosk, for user’s interface - see Additional file 2.

**Figure 3:**
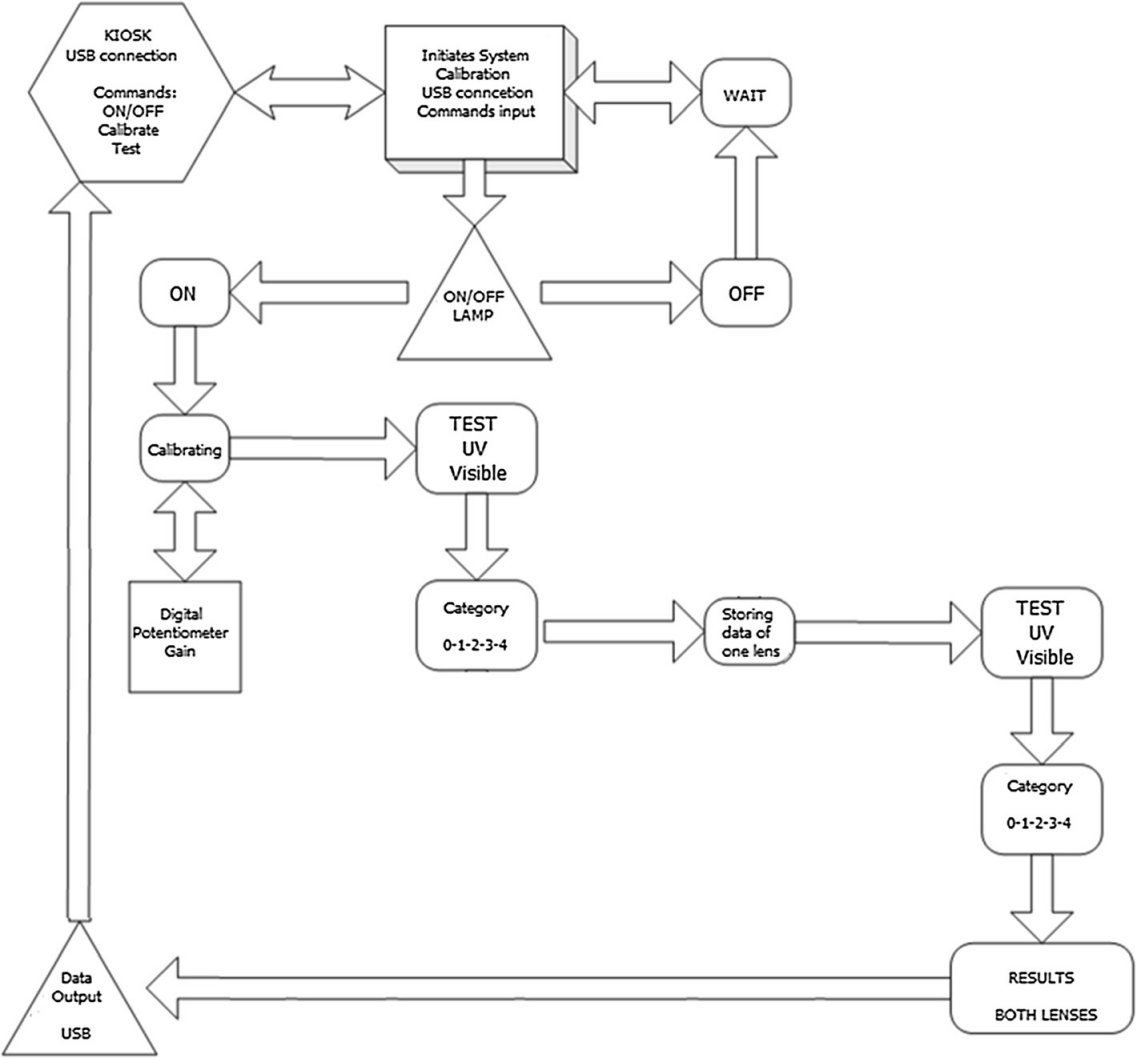
Block diagram of embedded software.

The embedded software was programmed using C language. It performs calibration of the system and controls the sequence for the transmittance measurements on the ultraviolet (280 – 400 nm) and visible (380 – 780 nm) ranges, providing the final report by comparing the data with boundary conditions of Table 1. Thus, a final report is presented for the user. The communication between the PC and microcontroller is done by USB protocol, which emulates a serial connection. The block diagram of the embedded software is shown in Figure 3.

**Figure 4:**
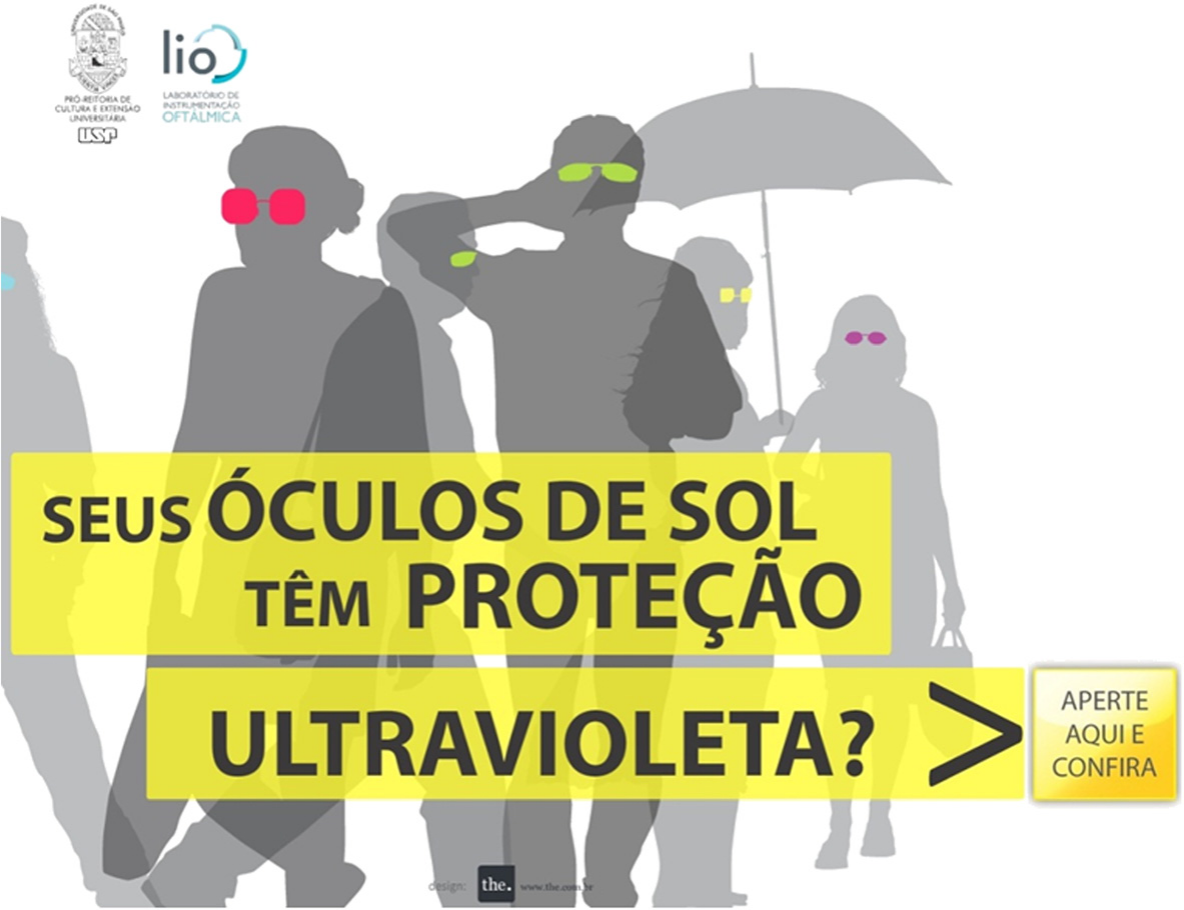
**Inviting screen for calling public attention for sunglasses UV protection self-testing: “ARE YOUR SUNGLASSES UV PROTECTED? > CHECK THEM HERE”.**

The controlling software of the kiosk was developed in Delphi. It features a total of 10 screens, providing information about proper protection for sunglasses and the harmful effects of ultraviolet radiation. It has an inviting and informative interface. The inviting screen is shown in Figure 4.

The software also establishes a communication with the microcontroller, sending commands to turn ON and turn OFF the lamp; calibrating the system; and performing the measurements.

The software is freely available for non-commercial purposes. It should run within the electronic and optical systems for working properly.

A database has been implemented for measured data storage at the kiosk as well as the survey responses for subsequent analysis. At the end of each test data from the ultraviolet protection, transmission of visible light and category measurements are saved as well as the report and survey responses.

The survey that is part of the system has been submitted to an Ethical Committee, so future sunglasses analysis may be available for the public and for the sunglasses standard committee, which is part of the activities of some of the authors of this work. Hence, the survey has been submitted to Ethical Committee - CONEP (Conselho Nacional de Ética em Pesquisa – National Consul of Ethics in Research) and has been approved under the registration number: 160.248 - CCAE: 02140312.5.0000.5504 at the Ethical Committee of CEP UFSCar. The study is being conducted in accordance with the provisions of the Declaration of Helsinki for experimentation involving human ethics.

Furthermore, the kiosk has a presence sensor, which is activated by the presence of someone passing by, calling the attention, by a recorded voice that says: “Are your sunglasses UV protected? Check them here!”

The sequenced interface screens guide the user for testing the sunglasses, as well as provide them information about each item to be tested. It also states previously to the tests that the machine is for sunglasses testing purposes only.

## Results

**Figure 5:**
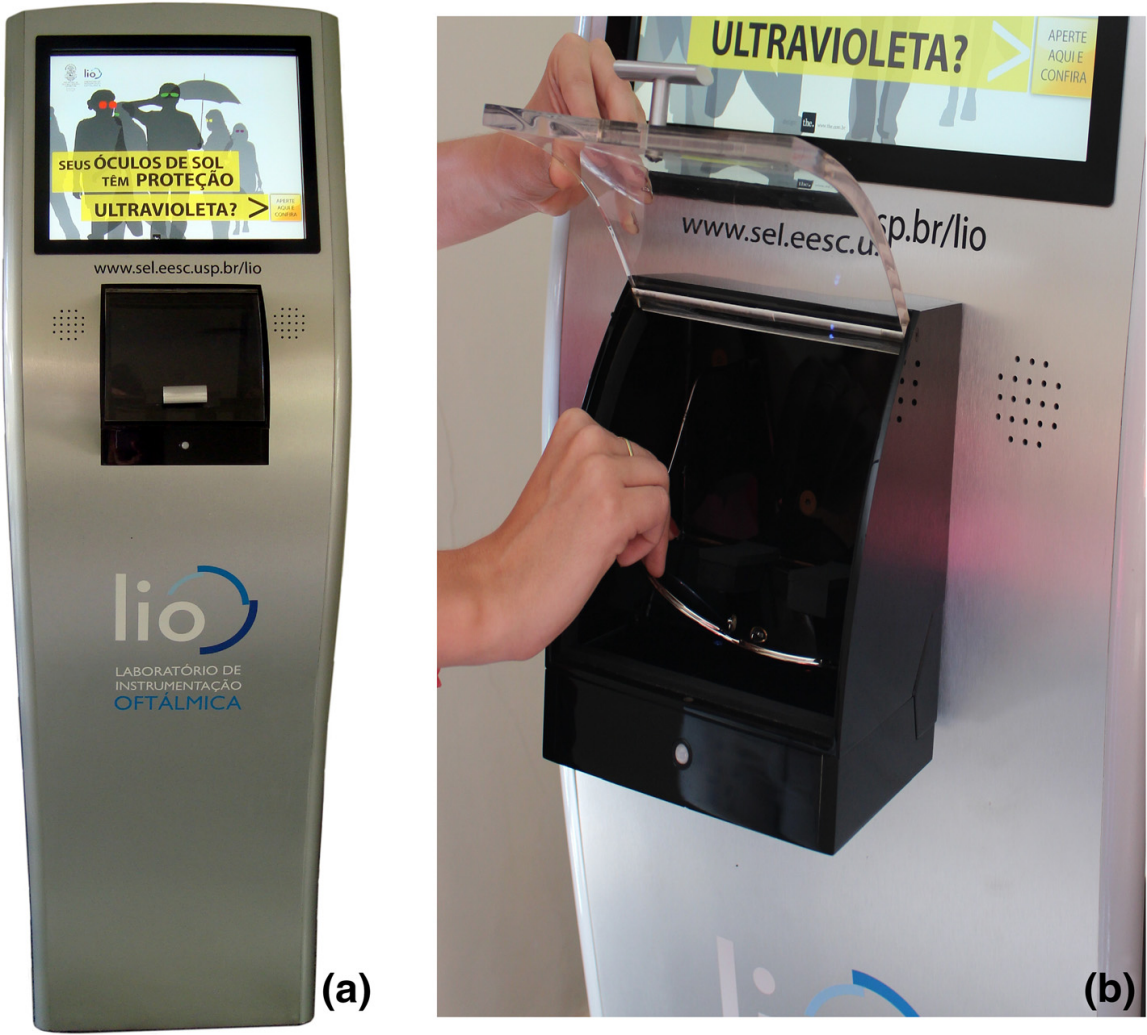
**Picture of the available system for the public. (a)** The self-service sunglasses testing kiosk; **(b)**; inserting sunglasses for testing.

The outcome of this system is a self-service sunglasses testing kiosk, which is presented in Figure 5. It shows the prototype - kiosk - and in detail, the slot to place the sunglasses for testing.

The 389 sunglasses that are part of the tests in this work were obtained from donation: 1, ABIOTICA – Brazilian Association of Optics Stores have donated 369 from apprehension by the Federal authorities of irregular trading of sunglasses on the streets); 20 were donated from signature sunglasses companies;

To test the system, its repeatability and accuracy, measurements were performed as described below.

### Repeatability

**Figure 6:**
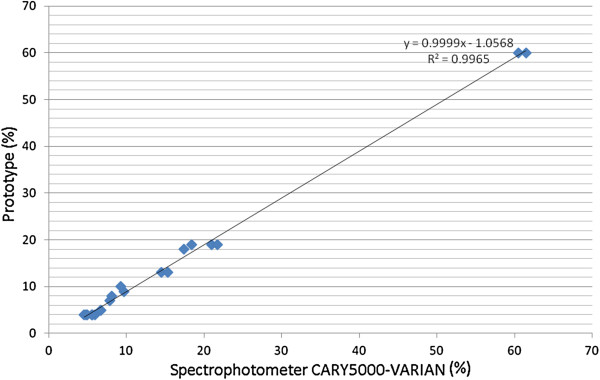
Repeatability measurements comparing the maximum and minimum values of different transmittances for the visible range.

The test consisted of performing repeated measurements of transmittance at different positions and angles within the device, for 10 different sunglasses (05 from ABIÓPTICA and 05 from the signature companies). Figure 6 shows the results obtained for visible light transmittance, emphasizing the values of maxima and minima obtained by the average transmittance values. The maximum difference obtained from the mean value for these measures was 3%. For UV transmittance, the sunglasses measures showed practically all 0% UV transmittance, and the maximum difference was 1% from the mean value.

### Benchmarking tests

Two tests were performed on each of the 20 lenses from 10 sunglasses in the developed equipment and the results were compared to spectrophotometric measurements.

**Figure 7:**
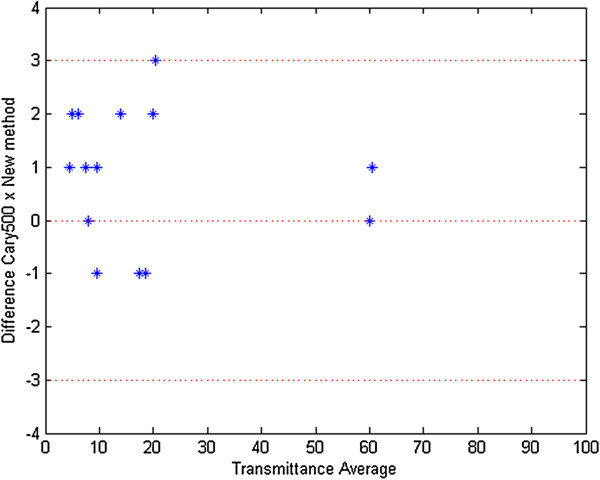
**Bland-Altman plot measurements of sunglasses tested on the prototype and on the spectrophotometer (visible range).**

Figure 7 shows the Bland-Altman plot for sunglasses tested on the prototype and on the spectrophotometer VARIAN CARY 5000 in the visible range. The transmittances of the UV range wavelengths for all the lenses were below 1%. The limit deviation for transmittance measurements shall be ± 3% absolute for the transmittance values of categories 0 to 3 and ± 30% relative to the stated value for the transmittance values of category 4 [10]. We have used the 3% limit in Figure 7 to determine the proximity between the two methods.

Furthermore, 45 additional sunglasses (35 from ABIÓPTICA and 10 from signature companies) - 90 lenses - have been tested and results show that there is a good correlation for the measurements of transmittance in the visible spectral range (r^2^ = 0.9999) and in the ultraviolet range (r^2^ = 0.9997).

In addition, the prototype has been tested with a calibration UV filter Model 81017 + 87066 from Newport and repeatability for the filter for 100 measurements is 99%.

There is a critical scenario when referring to provide a final report based on the limits of the standard (Table 1) which lies on the threshold between categories: the measurements in which the transmittance in the visible spectrum are coincident with the threshold values for changing category, namely: 8%, 18%, 43% or 80%. As tolerable by the standard that the accuracy is within ± 2% for the categories, the category indicated by the equipment and the spectrophotometer may present discrepancies. The standard provides only superposition of the categories 0, 1, 2 and 3 for the visible transmittance of ± 2% (absolute), which provides a region of 4 percentage points of transition, but does not clearly establishes the allowed values of transition to category 4 . It has been assumed accuracy of ± 1% (absolute) for categories 3 and 4.

It is worth noting that the problem becomes even greater when the threshold refers to categories 3 and 4, since in this situation the percentage of ultraviolet transmittance allowed is 1.0 τ_V_ and 0.5 τ_V_ for categories 3 and 4, respectively. This may infer a result of IMPROPER FOR USE, when in fact the sunglasses would be PROPER FOR USE. The reverse can also occur. In these cases, the device may indicate the wrong category.

To better illustrate the problem, consider hypothetically a pair of spectacles in which the lenses are tested on this developed prototype and has a transmittance of 9% (category 3), while in fact, spectrophotometric measurements indicate 7% (category 4).

In this particular case, the system measures the UVA and UVB radiation passing through the lens, indicating 4.5% UVA transmittance. These glasses are reported PROPER FOR USE for category 3 but should be actually IMPROPER for USE, because they belong to category 4. Category 4 is the only one in which the UVA transmittance should not exceed 0.5 τ_V_.

This is the only condition in which the error inherent in the system may indicate a false result, i.e., the only situation in which the system error affect the result is the visible range transmittance around 8%.

Therefore, for transmittance measurements in the visible range up to 7%, the equipment considers the threshold for UVA transmittance as being 1.0 τ_V_.

## Conclusions

A prototype has been built and it identifies the UV protection, according to Brazilian Standard NBR15111:2013, for non-corrective sunglasses according to the category of the lens, performing measurements of average light transmittance in the visible spectral region - 380 nm – 780 nm - and ultraviolet range - 280 – 400 nm.

This prototype may be an alternative for educating people and getting the public’s attention on the benefits of wearing UV protected sunglasses. The immediate clinical implication would be avoiding early cataract and possibly minor damages to the cornea that are still under discussion on literature.

The prototype has been exhibited for the public for free self-testing of their sunglasses. Stored data from survey and testing results are restricted to author of this work.

The public has already tested over 800 sunglasses. A small fraction - 20% - of them presented non-compliance, ie, IMPROPER USE. Most non-compliant - 93% - belong to category 2. Thus, the prototype in addition to educating the population about the importance of wearing protected sunglasses, has also allowed the public to have access to information about the quality of protection of their own sunglasses in an easy and free testing method.

Other meters can be found in the market, but they lack accuracy and correlation with measurements made with spectrophotometers.

However an imminent tendency towards building transmittance meters using LEDs can be observed. The light source suggested for use is the illuminant D65 standard, from CIE, but an assembly with LEDs appears to be an alternative in cost, size and low power consumption for portable meters.

## Competing interests

The authors declare that they have no competing interests.

## Authors’ contributions

MM carried out the development of the electronics for the visible range (category measurements) and participated on the electronics for the ultraviolet range; VACL carried out the development of the electronics for the UV range and also was responsible for the user’s interface software development; LV conceived of the study, carried out the optical development, participated in its design and coordination and drafted the manuscript. All authors read and approved the final manuscript.

## Supplementary Material

Additional file 1 Software for the microcontroller board. Click here for file

Additional file 2 Software for the communication of the PC, running Windows’ system, placed inside the kiosk, for user’s interface. Click here for file
